# Effects of endometrial injury on frozen‐thawed blastocyst transfer in hormone replacement cycles

**DOI:** 10.1002/rmb2.12031

**Published:** 2017-04-09

**Authors:** Yukiko Matsumoto, Shoji Kokeguchi, Masahide Shiotani

**Affiliations:** ^1^ Hanabusa Women's Clinic Kobe Japan

**Keywords:** blastocyst transfer, **e**ndometrium, endometrium sampler, injury, pregnancy rate

## Abstract

**Aim:**

To evaluate whether local endometrial injury would improve the pregnancy rates after frozen‐thawed blastocyst transfer in cycles with hormone replacement.

**Methods:**

Seventy‐seven patients who were aged <40 years with a history of at least three unsuccessful embryo transfers and who underwent frozen‐thawed blastocyst transfer during hormone replacement cycles between February and June, 2013 were studied. They were allocated into two groups based on the last digit of their clinical record number: an experimental group (n=22), excluding patients who withheld consent or who were unable to undergo treatment, and a control group (n=55). In the experimental group, the endometrium was scratched once with an endometrial sampler during the luteal phase of the cycle preceding the embryo transfer.

**Results:**

There was no significant difference in the baseline characteristics between the groups. The clinical pregnancy rate was 6% in the experimental group and 22% in the control group. Among these, the ongoing pregnancy rate was 100% in the experimental group and 50% in the control group.

**Conclusion:**

Endometrial injury might increase the pregnancy rates after frozen‐thawed blastocyst transfer and decrease the risk of miscarriage in patients with a history of unsuccessful embryo transfers.

## 1 Introduction

While treating infertile women with assisted reproductive technology, such as in vitro fertilization (IVF), the authors have observed that implantation failure can occur repeatedly in some women, even when transferring a good‐quality embryo. One possible cause for this is poor endometrial receptivity. Various approaches to addressing this problem have been explored and some recent studies have shown that local endometrial injury can improve both the implantation rates and pregnancy outcomes in patients with unexplained recurrent implantation failure.^1^, ^2^, ^3^, ^4^, ^5^, ^6^, ^7^, ^8^, ^9^

In 2003, a study reported that the sampling of the endometrium of IVF patients by using a biopsy catheter substantially increased their chances of conceiving during the subsequent IVF–embryo transfer cycles.^1^ Another study performed a systematic review and meta‐analysis in order to assess the efficiency of endometrial injury in participants with unexplained repeated implantation failure and found that local endometrial injury in the preceding ovarian stimulation cycles had a beneficial effect.^2^

However, those interventions mainly focused on fresh embryo transfers. Frozen‐thawed embryo transfers have been performed frequently with the aim of preventing the development of ovarian hyperstimulation syndrome and improving the pregnancy rate. In fact, the pregnancy rates of frozen‐thawed embryo transfer cycles have risen consistently, and in the past decade, have exceeded the pregnancy rate of fresh embryo transfer cycles.^10^

One study reported a negative impact of frozen‐thawed embryo transfer on pregnancy rates, but it only investigated cleavage‐stage embryos, not blastocysts, and not only patients with repeated implantation failure.^11^

To the best of the authors’ knowledge, no study has reported the use of induced endometrial injury in frozen‐thawed blastocyst transfer cycles in women with a history of repeated unsuccessful embryo transfers. Therefore, here is reported the effect of this procedure on frozen‐thawed blastocyst transfer in hormone replacement therapy.

## 2 Materials and Methods

One‐hundred‐and‐twenty‐four patients, aged <40 years old with a history of at least three unsuccessful embryo transfers who underwent blastocyst transfer during hormone replacement treatment between February and June, 2013 were enrolled in this study. All the patients underwent a hysteroscopy and hysterosalpingography prior to each embryo transfer cycle and all the abnormal findings were treated prior to the embryo transfer cycle. The patients with uterine morphological abnormalities were excluded from this study. The eligible patients were allocated to one of two groups, based on their clinical record number: a “scratch” group and an untreated control group.

In the scratch group, endometrial scratching was performed with an Endocyte^®^ sampler (Laboratoire CCD, Paris, France), as follows. The sampler was inserted through the cervical os and advanced gently along the shape of the uterus into the endometrial cavity. The outer catheter of the device was withdrawn to create suction, rotated three times in the same direction, and then pulled out. Scratching was performed once during the luteal phase of the cycle preceding the one that was used for the embryo transfer. Simultaneously, endometrial tissue was obtained from some patients with the endometrial sampler, processed for histology, stained with hematoxylin and eosin, and examined microscopically in order to evaluate how deeply this type of sampler invades the endometrium.

Transdermal estradiol, in combination with vaginal progesterone suppositories, was used in the hormone replacement cycles. The preparation of the endometrium started on day 2 with transdermal estradiol, with the doses escalating from 1.44 to 4.32 mg. The vaginal progesterone suppositories (1200 mg/day) were started on day 15. In all cases, it was checked that the endometrial thickness was ≥8 mm on that day. All the frozen‐thawed blastocyst transfers were performed on day 20 of the hormone replacement cycles. The endometrial thickness was measured again on the day of the blastocyst transfer.

The primary outcome measure in this study was the clinical pregnancy rate and the secondary outcome measure was the ongoing pregnancy rate. “Clinical pregnancy” was defined as the detection of a gestational sac in the uterus by ultrasound examination. “Ongoing pregnancy” was defined as a clinical pregnancy that continued after 12 weeks of gestation or resulted in a live birth.

The baseline characteristics of the two groups of patients are expressed as the mean±SD and as the number of patients. The scratch and control groups were compared by using the Student's *t* test for the continuous variables, while the chi‐square test with Yates’ correction was used to compare the categorical data, including the clinical and ongoing pregnancy rates. All the tests were two‐tailed and *P*<.05 was considered to be statistically significant.

All the patients provided written informed consent and the study's procedures were approved by the Institutional Review Board of Hanabusa Women's Clinic, Kobe, Japan.

## 3 Results

In total, 124 women were enrolled in the study. Of these, five participants in the scratch group withheld consent to participate in the study. In 23 cases, the scratching could not be performed during the appropriate period. A further eight women in the scratch group did not attend their clinic appointment for their procedure. Eleven women (four in the scratch group and seven in the control group) were excluded from the study because their second embryo transfer occurred during the intervention period. Therefore, the final scratch group comprised 22 participants and the control group comprised 55 participants. There was no significant difference in the baseline characteristics between the two groups (Table 1).

**Table 1:** Characteristics of the participants

Characteristic	Scratch group (n=22)	Control group (n=55)	*P*
Mean age (years)	35.50±2.89	35.62±2.72	NS
Duration of infertility (years)	2.78±1.93	3.10±2.41	NS
Type of subinfertility (N)			
Primary	18	35	NS
Secondary	4	20	
Number of transferred blastocysts	1.14±.35	1.07±.26	NS
Thickness of the endometrium (mm)	11.01±2.03	11.94±2.40	NS
Previous cycles of failed ETs	3.59±.91 (3–6)	4.13±2.04 (3–16)	NS
Causes of infertility, N (%)			
Male factor	6 (27.3)	9 (16.4)	NS
PCO	3 (13.6)	6 (10.9)	
POF	0 (.0)	1 (1.8)	
Endometriosis	1 (4.5)	1 (1.8)	
Tubal factor	8 (36.4)	30 (54.5)	
Unexplained	1 (4.5)	3 (5.5)	
Mixed	3 (13.6)	5 (9.1)	
Grade of the transferred blastocyst, N (%)			
<G4AB^a^	11 (50)	39 (70.9)	NS
≥G4AB^a^	11 (50)	16 (29.1)	

^a^Systems of Gardner and Schoolcraft blastocyst grading classification. ET, embryo transfer; NS, not significant; PCO, polycystic ovarian; POF, premature ovarian failure.

**Figure 1:**
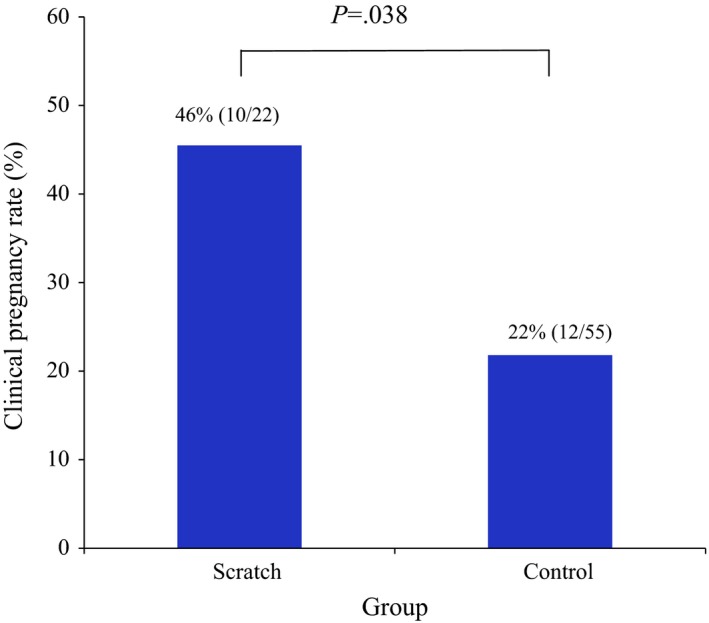
Clinical pregnancy rates in the scratch and control groups. The rate was significantly higher in the scratch group (46% vs 22%, respectively; *P*=.038)

**Figure 2:**
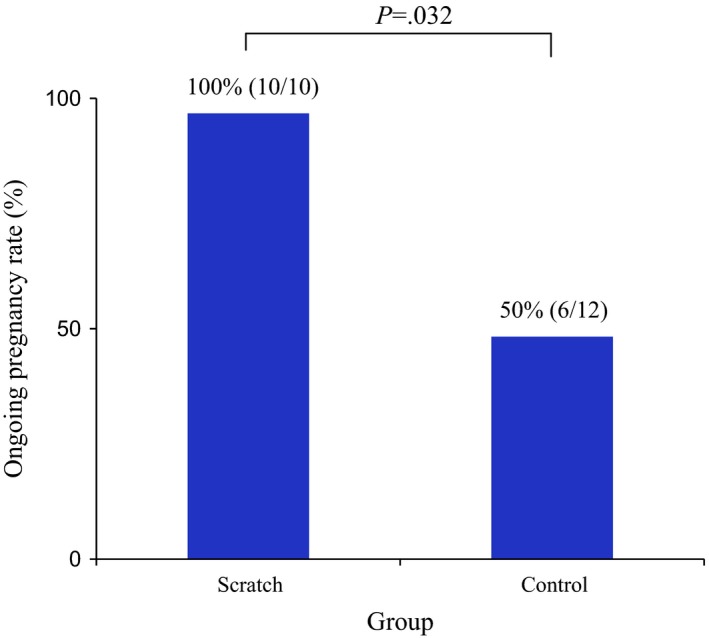
Ongoing pregnancy rate in the scratch and control groups. The rate was significantly higher in the scratch group (100% vs 50%, respectively; *P*=.032)

The clinical pregnancy rate was 10/22 (46%) in the scratch group and 12/55 (22%) in the control group (*P*=.038; Figure 1). Of these, the ongoing pregnancy rate was 10/10 (100%) in the scratch group, while it was 6/12 (50%, *P*=.032; Figure 2) in the control group.

**Figure 3:**
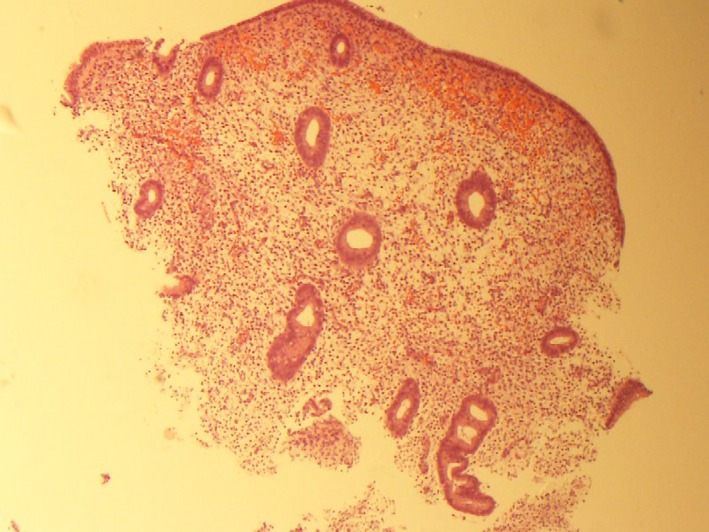
Endometrial tissue biopsy that was obtained from a patient by using an endometrial sampler, stained with hematoxylin and eosin (magnification ×40). The sampled tissues were distributed throughout the superficial part of the endometrium, limited to the functional zone, and did not reach the basilar zone

The endometrial tissues that were obtained by the sampler during the luteal phase showed only the superficial layer of the endometrium. The biopsied tissues were confined to the functional zone and did not extend into the basilar zone (Figure 3).

## 4 Discussion

In this study, artificial endometrial injury increased the pregnancy rate and decreased the abortion rate of women who underwent a frozen‐thawed blastocyst transfer following a history of repeated unsuccessful blastocyst transfers.

Concerning the effects of induced endometrial injury in frozen‐thawed embryo transfer cycles, a study reported a negative impact on the implantation and pregnancy rates.^10^ However, the cases included patients with a 0–1 previous embryo transfer, not only women with a history of repeated failure. The role of endometrial receptivity is unclear in women who are undergoing their first or second embryo transfer.

Several studies have reported that artificial endometrial injury improves the pregnancy rate.^1^, ^2^, ^3^, ^4^, ^5^, ^6^, ^7^, ^8^, ^9^ However, those reports varied in terms of the characteristics of the device that was used for injury, the degree of injury, and the timing of the intervention. Various devices have been used to injure the endometrium, such as hysteroscopes and biopsy catheters.^1^, ^3^, ^4^, ^5^, ^6^, ^7^, ^9^ In this study, an endometrial sampler was used that was considered to be an appropriate tool for inducing a mild endometrial injury. Moreover, such endometrial samplers are easy to obtain and manipulate; thus, the procedures could be standardized easily among physicians and clinics.

Moreover, the histological examination of the tissues that had been obtained from some of the patients revealed that scratching caused only a superficial injury, being confined to the functional layer and not reaching the basal layer. The authors believe that the depth of scratching would have been similar in all cases because the procedure was standardized and all scratches were created by the same physicians. These findings indicate that even superficial scratching with a sampler had a positive effect on pregnancy outcomes. However, it is unclear whether the depth of scratching would affect pregnancy outcomes.

In this study, the scratches were created during the luteal phase of the cycle preceding that used for embryo transfer for the convenience of the patients. One study stated that it is appropriate to take samples during the secretory phase of a cycle because injury‐induced decidualization is stimulated by progesterone.^1^ However, some studies have reported the effectiveness of injuring the endometrium during controlled ovarian hyperstimulation (COH) cycles.^4^, ^9^ One study reported that endometrial injury was performed during days 5‐22 of the COH cycle.^4^ Another study reported that the injury was performed during days 4‐7 of the COH cycle.^9^ In the current study, an injury was created once in the menstrual cycle. However, there is insufficient evidence as to how many such injuries are required in order to achieve beneficial results.^2^

Some mechanisms have been proposed by which the endometrial injury might increase endometrial receptivity and improve clinical pregnancy rates. One possibility is that decidualization might be induced in this way.^1^, ^4^, ^12^ Another is that wound healing elicits the secretion of cytokines and growth factors that might be beneficial for embryo implantation.^2^ Moreover, the reaction to endometrial injury might assist in synchronizing the embryos with the stage of the endometrium. A third possibility is the up‐regulation of gene expression. Taking a biopsy increases the expression of the gene encoding uroplakin Ib and other genes that are linked to uterine receptivity.^4^, ^13^

Interestingly, the favorable effects on endometrial receptivity were manifested in the cycle following the one involved in creating the injury. This long‐term effect might be associated with the activity of monocytes in the injured sites; these cells are long‐lived and can remain in some tissues for months.^7^

In conclusion, in the cycles involving frozen‐thawed blastocyst transfer, endometrial injury appears to improve the endometrial receptivity and increase the pregnancy rates in those women who are aged <40 years old and who have a history of at least two unsuccessful embryo transfers. The endometrial sampler that was used in this study is easy to use, the procedure could readily be standardized among physicians, and there is no obvious adverse side‐effect. The limitations of this study are the small number of participants and the incomplete nature of random allocation. Further studies to evaluate the effectiveness of induced endometrial injury on pregnancy outcomes with more participants and complete random allocation are needed.

## Disclosures

*Conflict of interest*: The authors declare no conflict of interest. *Human and Animal Rights*: All the procedures were followed in accordance with the standards of the responsible ethics committees on human experimentation (institutional and national) and with the Helsinki Declaration of 1964 and its later amendments. Informed consent was obtained from all the patients included in the study. This article does not contain any study with animal participants that was performed by any of the authors.
